# Stimulated Human Umbilical Cord Mesenchymal Stem Cells Enhance the Osteogenesis and Cranial Bone Regeneration through IL-32 Mediated P38 Signaling Pathway

**DOI:** 10.1155/2024/6693292

**Published:** 2024-03-13

**Authors:** Xiaru Zhang, Ying Zheng, Gang Wang, Yuanlin Liu, Yang Wang, Xueyi Jiang, Yueqing Liang, Xinfeng Zhao, Ping Li, Yi Zhang

**Affiliations:** ^1^Department of Experimental Hematology and Biochemistry, Beijing Institute of Radiation Medicine, Beijing 100085, China; ^2^Department of Stomatology, Beijing Chaoyang Hospital, Capital Medical University, Beijing 100020, China; ^3^Medical School of Chinese PLA, Beijing 100853, China; ^4^Laboratory of Nutrition and Development, Key Laboratory of Major Diseases in Children's Ministry of Education, Beijing Pediatric Research Institute, Beijing Children's Hospital, National Center for Children's Health, Capital Medical University, Beijing 100045, China

## Abstract

**Objective:**

Our previous study found that it could significantly increase the expression of IL32 after stimulating the human umbilical cord mesenchymal stem cells (S-HuMSCs). However, its role on the osteogenesis and cranial bone regeneration is still largely unknown. Here, we investigated the possible mechanism of this effect. *Material and Methods*. A series of experiments, including single-cell sequencing, flow cytometry, quantitative real-time polymerase chain reaction, and western blotting, were carried out to evaluate the characteristic and adipogenic–osteogenic differentiation potential of IL-32 overexpression HuMSCs (IL-32^high^HuMSCs) through mediating the P38 signaling pathway. Moreover, a rat skull bone defect model was established and treated by directly injecting the IL-32^high^HuMSCs to conduct its role on the cranial bone regeneration.

**Results:**

In total, it found that compared to HuMSCs, IL32 was significantly increased and promoted the osteogenic differentiation (lower expressions of PPAR*γ*, Adiponectin, and C/EBP*α*, and increased expressions of RUNX2, ALP, BMP2, OPN, SP7, OCN, and DLX5) in the S-HuMSCs (*P* < 0.05). Meanwhile, the enhanced osteogenic differentiation of HuMSCs was recovered by IL-32 overexpression (IL-32^high^HuMSCs) through activating the P38 signaling pathway, like as the S-HuMSCs (*P* < 0.05). However, the osteogenic differentiation potential of IL-32^high^HuMSCs was significantly reversed by the P38 signaling pathway inhibitor SB203580 (*P* < 0.05). Additionally, the HuMSCs, S-HuMSCs, and IL-32^high^HuMSCs all presented adipogenic–osteogenic differentiation potential, with higher levels of CD73, CD90, and CD105, and lower CD14, CD34, and CD45 (*P* > 0.05). Furthermore, these findings were confirmed by the rat skull bone defect model, in which the cranial bone regeneration was more pronounced in the IL-32^high^HuMSCs treated group compared to those in the HuMSCs group, with higher expressions of RUNX2, ALP, BMP2, and DLX5 (*P* < 0.05).

**Conclusion:**

We have confirmed that S-HuMSCs can enhance the osteogenesis and cranial bone regeneration through promoting IL-32-mediated P38 signaling pathway, which is proved that IL-32 may be a therapeutic target, or a biomarker for the treatment of cranial bone injuries.

## 1. Introduction

With the rapid development of economy and population aging, cranial bone injury has become a serious health issue with an ever-increasing prevalence, as it has estimated that in China, its occurrence has been nearly 100–200 per hundreds of thousands of people [1, 2]. Recently, the mainly existing treatments of cranial bone injury are included the autologous and allogeneic bone transplantation [3]. Otherwise, there are many disadvantages of these clinical usages for the limited sources, invasive, immune rejection, and insufficient osteogenic ability [4]. So in this condition, HuMSCs, as the common pluripotent stem cells, have become a seed candidate in the regenerative clinical medicine because of their easy separability, high moving trends, multidirectional differentiation potential, low immunogenicity, and noncytotoxicity [5]. Commonly, HuMSCs can differentiate into different precursor cells under the relevant conditioned media, in which it is balanced of adipogenic–osteogenic differentiation programs. Nowadays, a large number of clinical studies have shown that local transplantation of HuMSCs has a good improvement effect on the bone defects and osteoporosis through increasing the expressions of TGF-*β*1, Runx2, osteoblasts, Wnt10b, and so on [6, 7]. Moreover, the animal experiments also have proved that tail vein injection of HuMSCs can promote the reconstruction of cranial bone injury through increasing the alkaline phosphatase (ALP) activity and reduce the contents of triglyceride [8, 9]. Otherwise, there are still some studies that were obtained the different consequences, which point out that HuMSCs can not form the ectopic new bone in the subcutaneous tissues under the natural state [10, 11]. Therefore, it is very urgent and important to discuss the methods for improving the osteogenetic ability by the related postprocessing.

As we all known, the balances of adipogenic–osteogenic differentiation of HuMSCs can be affected by the microenvironment [1, 5]. Meanwhile, some scholars had found that pretreatment of HuMSCs with proinflammatory cytokines, as the stimulated HuMSCs (S-HuMSCs), could enhance their immunosuppressive properties [10]. However, no reports were discussed for the roles of S-HuMSCs on the osteogenesis and cranial bone injury in vivo and in vitro experiments. As demonstrated by our previous single-cell sequencing data, compared with the HuMSCs, S-HuMSCs lead to many aberrant expressions of related genes to enhance their biological functions and immune regulatory ability, in which there was a significant increase in the expression of IL-32 (HuMSCs) in the S-HuMSCs. Here, we found that IL-32, known as NK4, were contained in “RGD” motif-containing attachment sequence and it was a member of cytokines to induce the proinflammatory cytokines such as IL-1*β*, TNF-*α*, IL-6, and IL-8 [12, 13]. Additionally, a number of studies have revealed that actual participation of IL-32 depends on a series of downstream signaling activation events, as PI3K-AKT, NF-*κ*B, P38-mitogen-activated protein kinases (MAPK), ERK-MAPK, JNK-MAPK, and STAT1 pathways [14]. Moreover, it has also shown that when IL-32 was added to PBMC cultures, it could maintain with soluble receptor activator of RANKL. Although the numbers of newly generated osteoclasts were increased, the percentage of lacunar resorption was significantly decreased, suggesting a possible inhibitory effect of IL-32 on the osteoclast activation [15]. Meanwhile, some researches also have revealed the participation of IL-32 in the immune diseases, inflammatory reactions, and angiogenesis [16, 17]. However, IL-32, as an important regulatory factor in the S-HuMSCs, is involved in the osteogenesis and cranial bone injury, particularly the aggressiveness mediated by the immune microenvironment through the downstream signaling pathway, which is largely unknown. Therefore, this study aimed to verify the hypothesis that S-HuMSCs could enhance the osteogenesis and cranial bone regeneration by upregulating the IL-32/P38 signaling pathway.

## 2. Materials and Methods

### 2.1. Cell Preparation and Characteristics of HuMSCs and S-HuMSCs

The resuscitated HuMSCs (P3) from the laboratory were inoculated in the *α*-MEM medium containing 10% fetal bovine serum (FBS, Gibco, USA), 2 mM glutamine (Sigma, Germany), 100 U/mL penicillin (Sigma, Germany), and 100 mg/mL streptomycin (Sigma, Germany) in the T75 cell culture bottle (Corning, USA) [18]. Until the adherent HuMSCs reached a confluence of approximately 80%, the inflammatory factors as TNF-*α* (20 ng/mL, PeproTech, AF-315-01A) and IFN-*γ* (20 ng/mL, PeproTech, AF-315-05) were added to obtain the S-HuMSCs by stimulating more than 12 hr. Then, the morphology of HuMSCs and S-HuMSCs were observed under an inverted microscope when the degree of cell fusion reached 80%–90%. Meanwhile, they were phenotypically fixed, stained, and characterized by the fluorophore-conjugated monoclonal antibody permeabilization process, as the APC-anti-human CD14 monoclonal antibody (Invitrogen, 12-9952-41), APC-anti-human CD34 monoclonal antibody (Invitrogen, 17-0349-42), APC-anti-human CD45 monoclonal antibody (Invitrogen, 17-0459-42), PE-anti-human CD73 monoclonal antibody (Invitrogen, 12-0739-42), PE-anti-human CD90 monoclonal antibody (Invitrogen, 12-0909-42), and PE-anti-human CD105 monoclonal antibody (Invitrogen, 12-1057-42). All the characteristics of HuMSCs and S-HuMSCs were recorded by the flow cytometry using a FACScalibur system (Becton Dickinson) and analyzed by the FlowJo software [19, 20].

### 2.2. Adipogenic–Osteogenic Differentiation Potential of HuMSCs and S-HuMSCs

To identify the adipogenic–osteogenic differentiation potential, both the HuMSCs and S-HuMSCs were cultured with a complete *α*-MEM medium and related adipogenic (8 × 10^4^ cells/well in six-well plate, 10^−3^ mM dexamethasone, 0.5 mM isobutyl methylxanthine, 0.2 mM indomethacin, and 10 *μ*g/mL insulin; Sigma, Germany) and osteogenic inducers, respectively (3 × 10^4^ cells/well in six-well plate, 10^−7^ mM dexamethasone, 0.5 mM ascorbic acid, and 10 mM *β*-glycerol phosphate; Sigma, Germany) for 7 days, which was changed every 3 days. Meanwhile, the self-differentiated groups without the above inducers were as the controls. Moreover, the adipogenic differentiation potential of HuMSCs and S-HuMSCs were evaluated by Oil Red O staining (Sigma, Germany) and related gene expressions on the adipogenic differentiation (PPAR*γ*, Adiponectin, and C/EBP*α*). While their osteogenic differentiation capacity was assessed by ALP staining, alizarin red staining (ARS) and related gene expressions on the osteogenic differentiation (RUNX2, ALP, OPN, DLX5, SP7, OCN, and BMP2) [19, 20].

### 2.3. Single-Cell Sequencing of HuMSCs and S-HuMSCs

The samples of HuMSCs and S-HuMSCs (*n* = 3/group) were prepared in the cell suspensions and subjected to a series of multiple dilutions to make the cells under a single cell state. Then the whole mRNA was extracted and reverse transcribed into cDNA by the reverse transcriptase, and the gene expressions and immune repertoire data were obtained by the high-throughput sequencing technology according to the standard protocols by Shanghai OE Biotech. Co., Ltd. (Shanghai, China).

### 2.4. Lentiviral Transfection of IL-32^high^HuMSCs

The IL-32 lentiviral vectors were contained by the overexpressed IL-32 sequence with green fluorescent protein (GFP) and puromycin resistance gene, while the control vectors (NC) were the no-load plasmid with only the GFP and puromycin resistance gene. These were also constructed and harvested by the Genepharma (Shanghai, China). Exactly, the HuMSCs were seeded in the six-well plate at 1 × 10^5^ cells/well. Then they were respectively transferred by the IL-32 lentiviral and no-load plasmid vectors to respectively obtain the IL-32^high^HuMSCs and NC-HuMSCs (1 × 10^7^ TU/mL, MOI = 10) when the HuMSCs were grew to 60%, with the no lentivirus HuMSCs as the controls (HuMSCs). After 24 hr of transfer-ration, IL-32^high^HuMSCs and NC-HuMSCs were replaced with a complete *α*-MEM medium containing puromycin (2 mmol/L) for 48 hr, which then were changed into *α*-MEM medium without puromycin when the degrees of HuMSCs were reached to 90%. The transfection efficiency of IL-32^high^HuMSCs and NC-HuMSCs, as evaluating the contents of GFP were observed by the fluorescence microscope and flow cytometry. Furthermore, the transfection efficiency of target genes (IL-32) was detected by the quantitative real-time polymerase chain reaction (RT-PCR) and western blotting (WB).

### 2.5. Colony Forming Unit (CFU) and Proliferation of IL-32^high^HuMSCs

The HuMSC, NC-HuMSCs, and IL-32^high^HuMSCs were seeded in the six-well plate as 500 cells/well, and the complete *α*-MEM medium was changed every 3 days. After 10 days treatment, the medium was discarded and washed twice with phosphate buffered saline (PBS), which were then added and fixed with 1% paraformaldehyde for 15 min. They were afterwards rinsed twice with PBS and 1 mL crystal violet staining solution (Beyotime, China) was stained for 30 min. Then the numbers of colonies were counted by the Image J software. Moreover, the HuMSC, NC-HuMSCs, and IL-32^high^HuMSCs were also seeded in the 96-well plate as 2,000 cells/well with three duplicates, then 10 *μ*L CCK-8 (Dojindo, Japan) detection solution was added and detected by the microplate reader after the treatment for 9 days.

### 2.6. Animal Model of Cranial Bone Defect

Five 8-week-old Wistar rats were anesthetized by injecting 1% pentobarbital (0.6 mL/100 g), and two cranial bone lacunae were obtained using the dental drill (diameter: 5 mm) on two sides of midcranial suture. Then 5 × 10^5^ NC-HuMSCs (left lacunae) and IL-32^high^HuMSCs (right lacunae) were treated with the above cranial bone lacunae. At the 8^th^ week after the operation, the rats were sacrificed, then the skull was removed, fixed, and observed by the Micro-CT to observe the area of new bone formation. Meanwhile, the newly formed bone samples were taken to measure the expressions of genes on the osteogenic differentiation.

### 2.7. Effects of IL-32 on the MAPK Signaling Pathways

The protein expressions of MAPK signaling pathways such as P38, ERK and JNK, in the HuMSCs, NC-HuMSCs, and IL-32^high^HuMSCs were detected by WB method. Then the interactions between IL32 and P38 were measured by the CO-IP method [18]. Furthermore, the osteogenic differentiation capacity was assessed by ALP staining, ARS staining and related gene expressions on the osteogenic differentiation (RUNX2, ALP, and DLX5) after using the P38 signaling pathway inhibitor SB203580.

### 2.8. Quantitative Real-Time PCR

Total RNA was extracted from the HuMSC, S-HuMSCs, NC-HuMSCs, IL-32^high^HuMSCs, IL-32^high^HuMSCs + SB203580, and newly formed bones using the TRIzol Reagent (Invitrogen). And their cDNA samples were reverse transcribed by Transcript® One-Step gDNA Removal and cDNA Synthesis SuperMix (TransGen Biotech, China). The above genes involved on the IL-32, adipogenic (PPAR*γ*, Adiponectin, and C/EBP*α*) and osteogenic differentiation (RUNX2, ALP, BMP2, OPN, SP7, OCN, and DLX5) were determined by the RT-PCR (No. AQ101-03, TransStart® Green qPCR SuperMix, TransGen Biotech, China). The primers were shown in Table 1, while GAPDH was used as an internal reference gene and 2^−*ΔΔ*Ct^ was chosen to represent the mRNA expressions.

### 2.9. Western Blotting

After the cells and newly formed bones were collected, 200 *µ*L RIPA lysate (Beyotime, China) were added, resuspended, and lysed at 4°C for 30–40 min, and then they were centrifuged at 12,000x *g* at 4°C for 30 min. Their concentrations were determined by the BCA kits (Beyotime, China). Then 10% SDS-PAGE plates (20 ng/samples) were used to determine the expressions of PPAR*γ* (No. 16643-1-AP, Proteintech), C/EBP*α* (No. 8178S, Cell Signaling Technology), RUNX2 (No. ab14933, Abcam), BMP2 (No. AF5163, Affinity), ALP (No. DF12525, Affinity), DLX5 (No. PA5-101134, Invitrogen), P38 (No. 9212S, Cell Signaling Technology), P-P38 (No. 9211S, Cell Signaling Technology), ERK (No. 4370, Cell Signaling Technology), P-ERK (No. 3192, Cell Signaling Technology), JNK (No. 9252, Cell Signaling Technology), P-JNK (No. 9251, Cell Signaling Technology), and GAPDH (No. 5174 T, Cell Signaling Technology; 1 : 1,000).

### 2.10. Statistical Analysis

All statistical analyses were conducted using SPSS 21.0, while *α* level of 0.05 and effect coefficient of 0.90 were defined as the statistical differences. All values were expressed as mean ± standard deviation (SD). Then the comparisons between two groups were assessed by the Student's *t*-test, whereas multigroup comparisons were analyzed using one-way ANOVA.

## 3. Results

### 3.1. Characteristics of the S-HuMSCs

**Figure 1:**
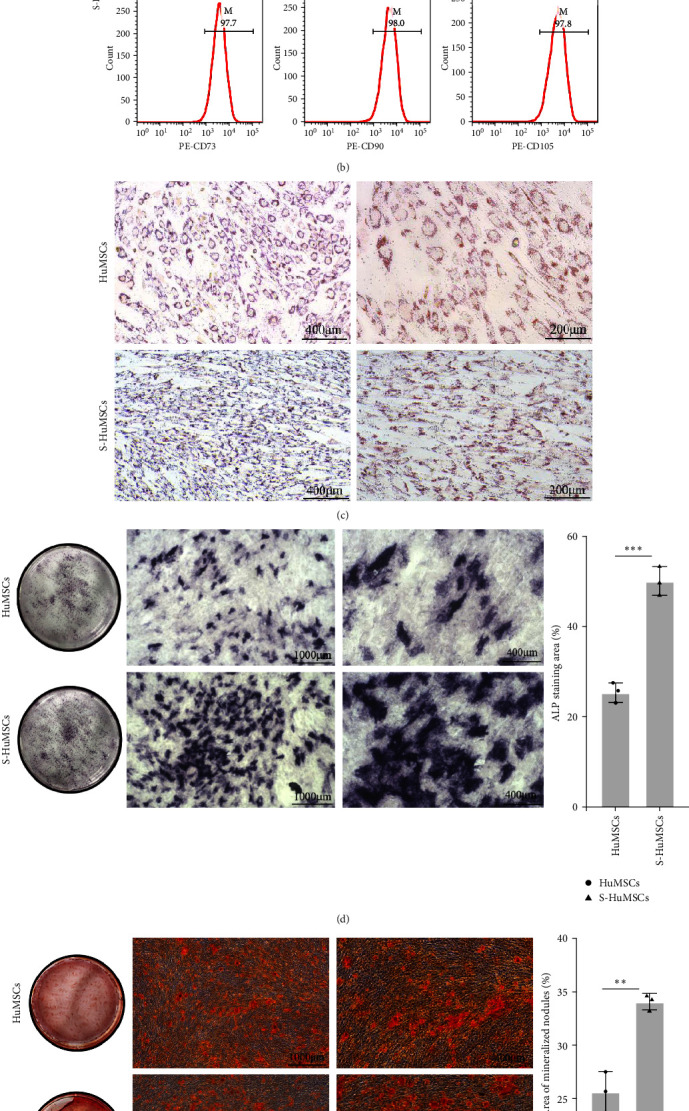
Osteogenic differentiation ability were enhanced under the stimulation by the inflammatory factors: (a) cell morphology; (b) antibody (CD73, CD90, CD105, CD14, CD34, and CD45) by the flow cytometry; (c) oil red o staining; (d) alkaline phosphatase staining; (e) Aalizarin red staining; (f) semiquantitative analysis of mineral compounds; (g) mRNA expression of genes on the adipogenic differentiation; (h) mRNA expression of genes on the osteogenic differentiation; (i) protein levels of PPAR*γ* and C/EBP*α*; and (j) protein levels of RUNX2, ALP, BMP2, and DLX5. All pooled data were represented as mean ± standard deviation (*n* = 3/group).  ^*∗*^*P* < 0.05,  ^*∗∗*^*P* < 0.01,  ^*∗∗∗*^*P* < 0.001.

Compared with the HuMSCs, the morphology of S-HuMSCs was similar (Figure 1(a), *P* > 0.05), with no significant differences in the purity of HuMSCs (CD73^+^, CD90^+^, CD105^+^, CD14^−^, CD34^−^, and CD45^−^Figure 1(b), *P* > 0.05).

### 3.2. Osteogenic Differentiation Ability were Enhanced under the Stimulation by the Inflammatory Factors (S-HuMSCs)

To determine the adipogenic and osteogenic differentiation potential of S-HuMSCs, Figure 1(c) showed that the numbers of lipid droplets were less in the S-HuMSCs than those in the HuMSCs, with significantly lower expressions of PPAR*γ*, Adiponectin, and C/EBP*α* (*P*  < 0.05; Figures 1(g) and 1(i)). Nevertheless, comparing with the HuMSCs, S-HuMSCs could enhance the osteogenic differentiation potential, which was confirmed by the larger staining areas through both the ALP (Figure 1(d)) and ARS methods (Figures 1(e) and 1(f)). Moreover, the expressions of key transcription factors on the osteogenic differentiation (RUNX2, ALP, OPN, DLX5, SP7, OCN, and BMP2) were significantly increased (*P* < 0.05; Figures 1(h) and 1(j)).

### 3.3. IL-32 was Highly Expressed in the S-HuMSCs

**Figure 2:**
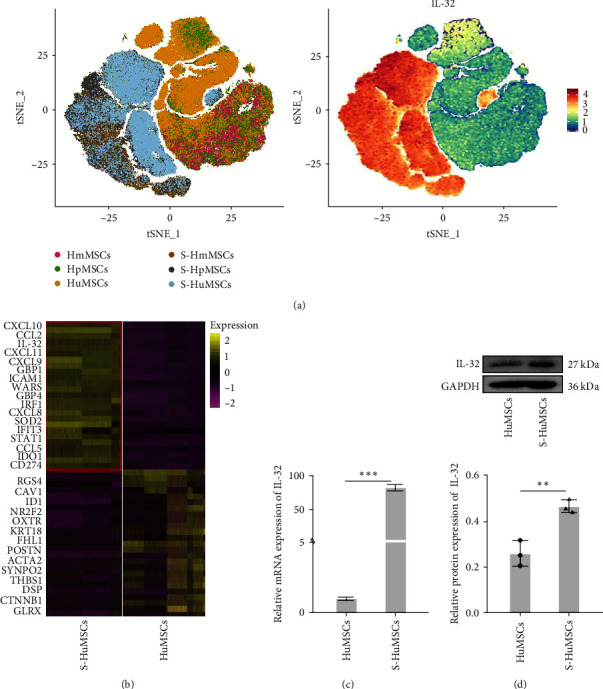
IL-32 was highly expressed in the S-HuMSCs by the single-cell sequencing: (a) and (b) differential gene expression profile between the HuMSCs and S-HuMSCs; (c) mRNA expressions of IL-32; and (d) protein levels of IL-32. All pooled data were represented as mean ± standard deviation (*n* = 3/group).  ^*∗*^*P* < 0.05,  ^*∗∗*^*P* < 0.01,  ^*∗∗∗*^*P* < 0.001.

As shown in Figures 2(a) and 2(b), the expressions of IL-32 were specifically higher in the S-HuMSCs than those in the HuMSCs by the single-cell sequencing (*P* < 0.05), which was also verified by RT-PCR (Figure 2(c)) and WB (Figure 2(d); *P* < 0.05).

### 3.4. IL-32^high^HuMSCs Could Promote the Osteogenesis and Cranial Bone Regeneration

**Figure 3:**
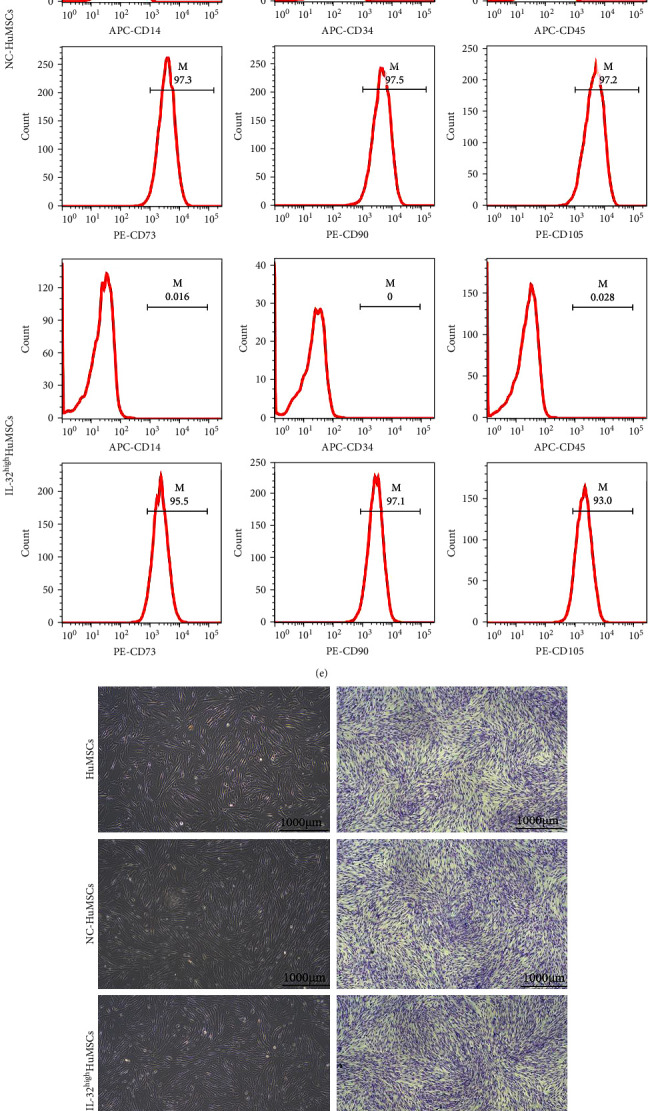
Transfection efficiency and characteristic of IL-32^high^HuMSCs: (a) and (b) transfection efficiency by the fluorescence microscope and flow cytometry respectively; (c) mRNA expressions of IL-32; (d) protein levels of IL-32; (e) antibody (CD73, CD90, CD105, CD14, CD34, and CD45) by the flow cytometry; (f) cell morphology; (g) colony formation rate; and (h) proliferation ability. All pooled data were represented as mean ± standard deviation (*n* = 3/group).  ^*∗*^*P* < 0.05,  ^*∗∗*^*P* < 0.01,  ^*∗∗∗*^*P* < 0.001.

As shown in Figures 3(a) and 3(b), the transfection efficiency was more than 90%, with no significant differences between the NC-HuMSCs and IL-32^high^HuMSCs. Meanwhile, the expressions of IL-32 were much higher in the IL-32^high^HuMSCs than those in the HuMSCs and NC-HuMSCs (*P* < 0.05; Figures 3(c) and 3(d)), with no changes on the characteristic of purity, self-renewal, and proliferation (*P* > 0.05; Figure 3(e)–3(h)).

**Figure 4:**
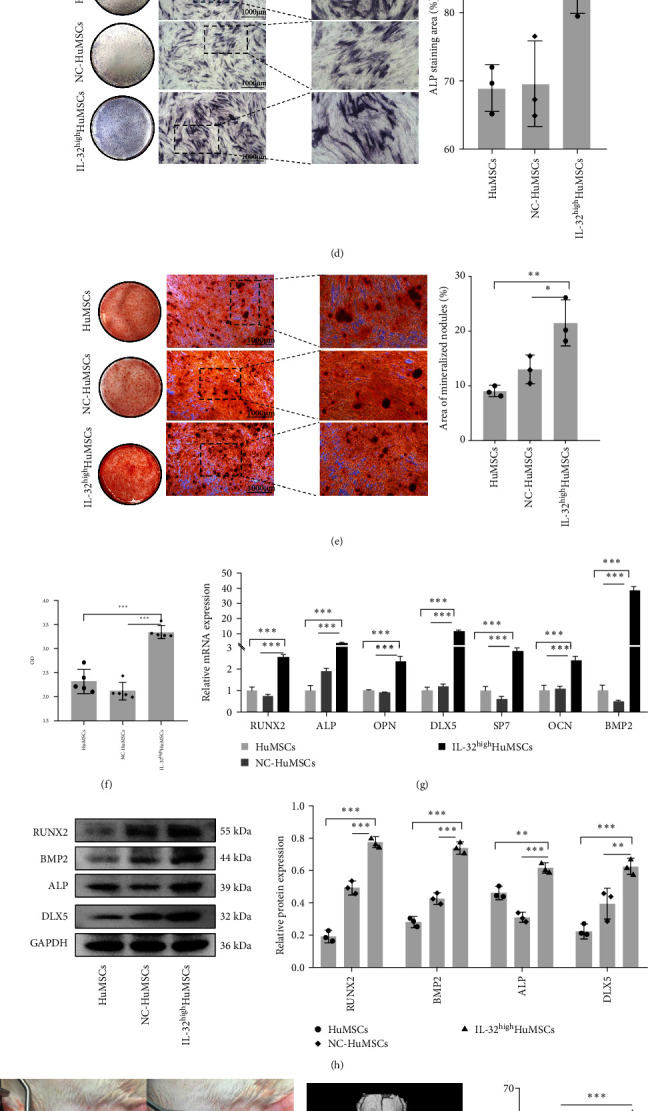
IL-32^high^HuMSCs could promote the osteogenesis and cranial bone regeneration: (a) lipid droplets by the oil red o staining; (b) mRNA expression of genes on the adipogenic differentiation; (c) protein levels of PPAR*γ* and C/EBP*α*; (d) alkaline phosphatase staining; (e) alizarin red staining; (f) semiquantitative analysis of mineral compounds; (g) mRNA expression of genes on the osteogenic differentiation; (h) protein levels of RUNX2, ALP, BMP2, and DLX5; (i) surgical procedure to create a skull defect; (j) Micro-CT of three-dimensional reconstruction and areas of new cranial bone regeneration; and (k) expressions of osteogenesis related genes in the new cranial bone. All pooled data were represented as mean ± standard deviation (SD, *n* = 3/group).  ^*∗*^*P* < 0.05,  ^*∗∗*^*P* < 0.01,  ^*∗∗∗*^*P* < 0.001.

Simultaneously, we then discussed the roles of IL-32 on the adipogenic and osteogenic differentiation potential. As shown in Figure 4(a), the numbers of lipid droplets in the IL-32^high^HuMSCs were significantly less than those in the HuMSCs and NC-HuMSCs groups, with the significant decreases on the gene expressions of PPAR*γ*, Adiponectin, and C/EBP*α* (*P*  < 0.05; Figures 4(b) and 4(c)). Otherwise, the results from ALP, ARS, and mineralized nodules were significantly higher in the IL-32^high^HuMSCs than those in the HuMSCs and NC-HuMSCs (*P* < 0.05); Figure 4(d)–4(f)). Meanwhile, the expressions of related key transcriptions on the osteogenic differentiation were significantly increased in the IL-32^high^HuMSCs (*P* < 0.05; Figures 4(g) and 4(h)). Furthermore, it was also confirmed by the animal model of cranial bone defect using the Micro-CT (*P* < 0.05; Figure 4(i)), which was accompanied by the significantly larger areas of new bone formation in the IL-32^high^HuMSCs than those in the NC-HuMSCs (*P* < 0.05), with the higher expressions of RUNX2, ALP, BMP2, and DLX5 (*P* < 0.05; Figure 4(k)).

### 3.5. IL-32 Promoted the Osteogenic Differentiation of HuMSCs through Activating the P38 Signaling Pathway

**Figure 5:**
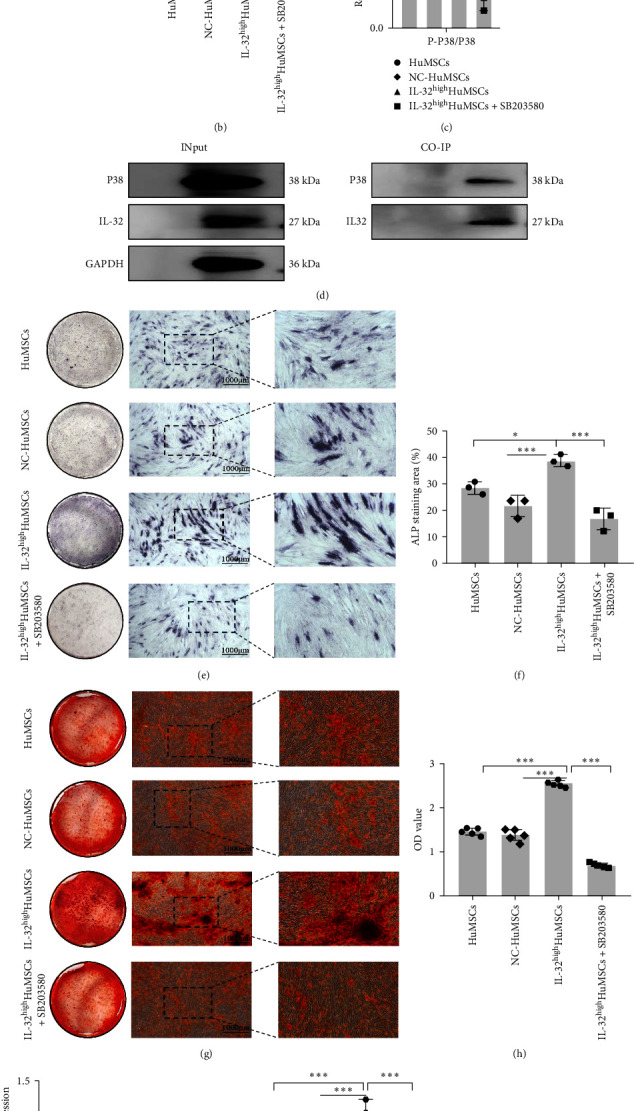
IL-32 promoted the osteogenic differentiation of S-HuMSCs through the MAPK-P38 pathway: (a) expressions of MAPK pathways, as the P38, ERK, and JNK; (b) and (c) protein levels of P-P38 and P38 after the treatment with SB203580; (d) INput and CO-IP assay between the IL-32 and P38; (e) and (f) alkaline phosphatase staining; (g) and (h) alizarin red staining and numbers of mineralized nodules; (i) mRNA expressions of RUNX2, ALP, and DLX5; and (j) and (k) protein levels of RUNX2, ALP, and DLX5. All pooled data were represented as mean ± standard deviation (SD, *n* = 3/group).  ^*∗*^*P* < 0.05,  ^*∗∗*^*P* < 0.01,  ^*∗∗∗*^*P* < 0.001.

As shown in Figure 5(a), the expressions of P-P38 were higher in the IL-32^high^HuMSCs than those in the HuMSCs and NC-HuMSCs (*P* < 0.05), with no significant differences of ERK and JNK pathways, which could be obviously reduced by inhibiting the P38 pathway with SB203580 (*P* < 0.05; Figure 5(b)). Moreover, both CO-IP and INput assay confirmed there were significant interactions between the IL-32 and P38 (*P* < 0.05; Figures 5(c) and 5(d)). Additionally, the osteogenic differentiation potential was significantly reduced in the IL-32^high^HuMSCs with SB203580 treatment, with smaller staining areas and mineralized nodules (*P* < 0.05; Figure 5(e)–5(h)), and lower expressions of RUNX2, ALP, and DLX5 (*P* < 0.05; Figure 5(i)–5(k)).

## 4. Discussion

Bone healing and regeneration, including the cranial bone, may be affected by the immune microenvironment, vascularization, and so on [21, 22]. Recently, many researches have proved this phenomenon. For example, Karnes et al. [23] had found that delayed endochondral and intramembranous osteogenesis was shown among the TNF*α*-receptor-deficient mice. Ishikawa et al. [24] also observed that CCL2-deficient mice could decrease the fracture healing and diminish the callus formation by reducing the infiltration of macrophages, vascularization, and MSCs. Besides, the interactions between immune microenvironment and HuMSCs differentiation can regulate the bone regeneration, in which HuMSCs play their roles on regulating the polarization of helper T cells, reducing the activation and proliferation of T cells, inhibiting of M1 macrophages activation, and inducing the generation of M2 macrophages through secreting the cytokines, such as PGE-2, TGF-*β*1, IL-6, HGF, MCP-1, and COX-2 under the inflammatory state [5–7]. Then, it had been proposed that MSCs could be pretreated with the proinflammatory cytokines (IFN-*γ* and TNF-*α*; S-HuMSCs) to enhance their immunosuppressive properties to promote the bone healing [25–28]. Moreover, Cai et al. [29] found that the immune MSCs were determined to be close with other cell subsets in the cell communication analysis, including the adipogenic–osteogenic differentiation programs. So far, Lu et al. [30] had reported that TNF-*α* pretreated human AT-MSCs could promote the proliferation and osteogenic differentiation of human primary osteoblasts. However, the limited source of BMSCs and AT-MSCs, and high incidences of related complications affected their wide application on the bone defects [6, 31]. Therefore, it is particularly important to discuss the roles of S-HuMSCs on the osteogenic differentiation, which was consistent with our conclusion.

As a unique cytokine, IL-32 can switch its roles between the proinflammatory and anti-inflammatory programs on many diseases as Crohn's disease, rheumatoid arthritis, chronic obstructive pulmonary disease, ulcerative colitis, psoriasis, atopic dermatitis, and tuberculosis and so on by producing the multiple isoforms through the alternative splicing [32–35]. In addition, other researchers had shown that IL-32 could participate in the airway inflammation by inducing the TNF-*α* [36]. So IL-32 has an important role on promoting inflammatory responses and inducing the differentiation of monocytes into the macrophage-like cells by arising CD11b, CD14, and CD44, which were the differentiation markers of macrophages [33, 35]. Moreover, many studies had proved that IL-32 could stimulate the osteogenesis and bone remodeling in vitro and in vivo [37, 38]. The current study further explored the mechanisms by the S-HuMSCs contributing to the easily osteogenesis. Our previous study suggested that IL-32, GBP2, CD200, PTX3, CHI3L1, and so on were highly expressed in the S-HuMSCs than those in the HuMSCs, in which the induction of IL-32 might be one mechanism to promote the osteogenic differentiation. So our research confirmed that S-HuMSCs could promote the osteogenesis and cranial bone regeneration by upregulating the expressions of IL-32. However, the osteogenesis mechanism of IL-32 is still unclear. Previous studies had shown that IL-32 could augment the productions of proinflammatory cytokines, such as TSLP, IL-1*β*, TNF*α*, IL-8, and IL-6, through p38-MAPK, NF-kB, and caspase-1 activation. Besides, the exosomes derived from the MSCs could promote the proliferation of osteoblasts through the P38-MAPK pathway by the osteoinductive ligand BMP2, Wnt signal, parathyroid hormone (PTH) and, etc. [39, 40]. In summary, IL-32 not only induces the production of inflammatory cytokines, but also activates the typical signaling pathways, mainly the P38-MAPK. In this study, we also verified that the P-P38 in IL-32^high^HuMSCs was significantly increased and osteogenic ability was significantly enhanced, which could be decreased when they were treated with SB203580, as a P38-MAPK pathway inhibitor.

## 5. Conclusion

In conclusion, our findings have confirmed that S-HuMSCs can enhance the osteogenesis and cranial bone regeneration through promoting IL-32-mediated P38 signaling pathway, which is proved that IL-32 may be a therapeutic target, or a biomarker for the mechanism of bone formation in the treatment of cranial bone injuries.

## Figures and Tables

**Table 1:** Primers of the selected genes.

Genes	Forward primer	Revised primer
IL32	AGAATCAGGACGTGGACAG	TAGAGGAGTGAGCTCTGGG
PPAR*γ*	GACCACTCCCACTCCTTTGA	ATTCAATTGCCATGAGGGAG
C/EBP*α*	CTGACCAGTGACAATGACC	CCTTGACCAAGGAGCTCTC
Adiponectin	TCACCCAAGCAACAAAGTC	AAAGACCAACCAGATGCAG
RUNX2	TTATTCTGCTGAGCTCCGG	GTGAAACTCTTGCCTCGTC
ALP	CAGAAGAAGGACAAACTGGG	ATTGTATGTCTTGGACAGAGC
DLX5	CTACGCTAGCTCCTACCAC	GTCACTTCTTTCTCTGGCTG
BMP2	CCACCATGAAGAATCTTTGGA	TGATAAACTCCTCCGTGGG
OPN	CCATACCAGTTAAACAGGCTG	TCAGGGTTTAGCCATGTGG
SP7	ACCCACCTCAGGCTATGCTA	TGCCCCCATATCCACCACTA
OCN	CTTTGTGTCCAAGCAGGAG	CTCCCAGCCATTGATACAG
GAPDH	TCAAGATCATCAGCAATGCC	CGATACCAAAGTTGTCATGGA

## Data Availability

The data and materials used and analyzed during the current study are available from the corresponding author on reasonable requests.

## References

[1] Qing-Bin Z., Zhao-Qiang Z., Dan C., Yan Z. (2013). Epidemiology of maxillofacial injury in children under 15 years of age in southern China. *Oral Surgery, Oral Medicine, Oral Pathology and Oral Radiology*.

[2] Wang J., Han F., Zhao Q. (2018). Clinicopathological characteristics of traumatic head injury in juvenile middle-aged and elderly individuals. *Medical Science Monitor*.

[3] Reis C., Gospodarev V., Reis H. (2017). Traumatic brain injury and stem cell: pathophysiology and update on recent treatment modalities. *Stem Cells International*.

[4] Trávníčková M., Bačáková L. (2018). Application of adult mesenchymal stem cells in bone and vascular tissue engineering. *Physiological Research*.

[5] Hu Z., Jiang Z., Meng S., Liu R., Yang K. (2023). Research progress on the osteogenesis-related regulatory mechanisms of human umbilical cord mesenchymal stem cells. *Stem Cell Reviews and Reports*.

[6] Zhao J., Meng H., Liao S. (2022). Therapeutic effect of human umbilical cord mesenchymal stem cells in early traumatic osteonecrosis of the femoral head. *Journal of Orthopaedic Translation*.

[7] Yaghoubi Y., Movassaghpour A. A., Zamani M., Talebi M., Mehdizadeh A., Yousefi M. (2019). Human umbilical cord mesenchymal stem cells derived-exosomes in diseases treatment. *Life Sciences*.

[8] Hu Y., He Y., Fang J. (2022). Wnt10b-overexpressing umbilical cord mesenchymal stem cells promote fracture healing via accelerated cartilage callus to bone remodeling. *Bioengineered*.

[9] Qiu C., Zhou J.-L., Deng S., Long L.-S., Peng H. (2021). Protective effect of human umbilical cord mesenchymal stem cells in glucocorticoid-induced osteonecrosis of femoral head. *Current Medical Science*.

[10] Song W., Zhao L., Gao Y. (2023). Dual growth factor-modified microspheres nesting human-derived umbilical cord mesenchymal stem cells for bone regeneration. *Journal of Biological Engineering*.

[11] Zheng Y., Yao H., Yang K. (2018). SFRP5 inhibits BMP9-induced osteogenic differentiation of human umbilical cord mesenchymal stem cells in vitro. *Chinese Journal of Biological Engineering*.

[12] Calabrese F., Baraldo S., Bazzan E. (2008). IL-32, a novel proinflammatory cytokine in chronic obstructive pulmonary disease. *American Journal of Respiratory and Critical Care Medicine*.

[13] Wen S., Hou Y., Fu L. (2019). Cancer-associated fibroblast (CAF)-derived IL32 promotes breast cancer cell invasion and metastasis via integrin *β*3-p38 MAPK signalling. *Cancer Letters*.

[14] Yousif N. G., Al-amran F. G., Hadi N., Lee J., Adrienne J. (2013). Expression of IL-32 modulates NF-*κ*B and p38 MAP kinase pathways in human esophageal cancer. *Cytokine*.

[15] Mabilleau G., Sabokbar A. (2009). Interleukin-32 promotes osteoclast differentiation but not osteoclast activation. *PLoS ONE*.

[16] Nold-Petry C. A., Nold M. F., Zepp J. A., Kim S.-H., Voelkel N. F., Dinarello C. A. (2009). IL-32-dependent effects of IL-1beta on endothelial cell functions. *Proceedings of the National Academy of Sciences*.

[17] Lee E.-J., Kim S.-M., Choi B. (2017). Interleukin-32 gamma stimulates bone formation by increasing mir-29a in osteoblastic cells and prevents the development of osteoporosis. *Scientific Reports*.

[18] Liu W., Yuan F., Bai H. (2022). hUC-MSCs attenuate acute graft-versus-host disease through Chi3l1 repression of Th17 differentiation. *Stem Cells International*.

[19] Li P., Wang Y., Li P. (2022). Maternal inappropriate calcium intake aggravates dietary-induced obesity in male offspring by affecting the differentiation potential of mesenchymal stem cells. *World Journal of Stem Cells*.

[20] Liu W., Zhou N., Liu Y. (2021). Mesenchymal stem cell exosome-derived miR-223 alleviates acute graft-versus-host disease via reducing the migration of donor T cells. *Stem Cell Research & Therapy*.

[21] He J., Chen G., Liu M. (2020). Scaffold strategies for modulating immune microenvironment during bone regeneration. *Materials Science and Engineering: C*.

[22] Xiong Y., Mi B.-B., Lin Z. (2022). The role of the immune microenvironment in bone, cartilage, and soft tissue regeneration: from mechanism to therapeutic opportunity. *Military Medical Research*.

[23] Karnes J. M., Daffner S. D., Watkins C. M. (2015). Multiple roles of tumor necrosis factor-alpha in fracture healing. *Bone*.

[24] Ishikawa M., Ito H., Kitaori T. (2014). MCP/CCR2 signaling is essential for recruitment of mesenchymal progenitor cells during the early phase of fracture healing. *PLoS ONE*.

[25] Noronha N. C., Mizukami A., Caliári-Oliveira C. (2019). Priming approaches to improve the efficacy of mesenchymal stromal cell-based therapies. *Stem Cell Research & Therapy*.

[26] de Witte S. F. H., Franquesa M., Baan C. C., Hoogduijn M. J. (2016). Toward development of iMesenchymal stem cells for immunomodulatory therapy. *Frontiers in Immunology*.

[27] Lin T., Pajarinen J., Nabeshima A. (2017). Preconditioning of murine mesenchymal stem cells synergistically enhanced immunomodulation and osteogenesis. *Stem Cell Research & Therapy*.

[28] Philipp D., Suhr L., Wahlers T., Choi Y.-H., Paunel-Görgülü A. (2018). Preconditioning of bone marrow-derived mesenchymal stem cells highly strengthens their potential to promote IL-6-dependent M2b polarization. *Stem Cell Research & Therapy*.

[29] Cai S., Fan C., Xie L. (2022). Single-cell RNA sequencing reveals the potential mechanism of heterogeneity of immunomodulatory properties of foreskin and umbilical cord mesenchymal stromal cells. *Cell & Bioscience*.

[30] Lu Z. F., Wang G. C., Dunstan C. R. (2013). Activation and promotion of adipose stem cells by tumour necrosis factor-*α* preconditioning for bone regeneration. *Journal of Cellular Physiology*.

[31] Gimble J. M., Zvonic S., Floyd Z. E., Kassem M., Nuttall M. E. (2006). Playing with bone and fat. *Journal of Cellular Biochemistry*.

[32] Heinhuis B., Plantinga T. S., Semango G. (2016). Alternatively spliced isoforms of IL-32 differentially influence cell death pathways in cancer cell lines. *Carcinogenesis*.

[33] Kitayama N., Otsuka A., Nonomura Y., Nakashima C., Honda T., Kabashima K. (2017). Decrease in serum IL-32 level in patients with atopic dermatitis after cyclosporine treatment. *Journal of the European Academy of Dermatology and Venereology*.

[34] Meyer N., Christoph J., Makrinioti H. (2012). Inhibition of angiogenesis by IL-32: possible role in asthma. *Journal of Allergy and Clinical Immunology*.

[35] Lee Y. S., Han S.-B., Ham H. J. (2020). IL-32*γ*suppressed atopic dermatitis through inhibition of miR-205 expression via inactivation of nuclear factor-kappa B. *Journal of Allergy and Clinical Immunology*.

[36] Li D., Chen D., Zhang X. (2015). c-Jun N-terminal kinase and Akt signalling pathways regulating tumour necrosis factor-*α*-induced interleukin-32 expression in human lung fibroblasts: implications in airway inflammation. *Immunology*.

[37] Joosten L. A. B., Netea M. G., Kim S.-H. (2006). IL-32, a proinflammatory cytokine in rheumatoid arthritis. *Proceedings of the National Academy of Sciences*.

[38] Spanjer E. C. K., Bittermann G. K. P., van Hooijdonk I. E. M., Rosenberg A. J. W. P., Gawlitta D. (2017). Taking the endochondral route to craniomaxillofacial bone regeneration: a logical approach?. *Journal of Cranio-Maxillofacial Surgery*.

[39] Ni S., Xiong X. B., Ni X. Y. (2020). MgCl2 promotes mouse mesenchymal stem cell osteogenic differentiation by activating the p38/Osx/Runx2 signaling pathway. *Molecular Medicine Reports*.

[40] Chan Y.-H., Ho K.-N., Lee Y.-C. (2022). Melatonin enhances osteogenic differentiation of dental pulp mesenchymal stem cells by regulating MAPK pathways and promotes the efficiency of bone regeneration in calvarial bone defects. *Stem Cell Research & Therapy*.

